# Using seminatural and simulated habitats for seed germination ecology of banana wild relatives

**DOI:** 10.1002/ece3.8152

**Published:** 2021-10-11

**Authors:** Simon Kallow, Katrijn Quaghebeur, Bart Panis, Steven B. Janssens, John Dickie, Lavernee Gueco, Rony Swennen, Filip Vandelook

**Affiliations:** ^1^ Royal Botanic Gardens Kew Millennium Seed Bank Ardingly UK; ^2^ Department of Biosystems Katholieke Universiteit Leuven Leuven Belgium; ^3^ Meise Botanic Garden Meise Belgium; ^4^ Alliance of Bioversity International and the International Center for Tropical Agriculture Leuven Belgium; ^5^ Biology Department Katholieke Universiteit Leuven Leuven Belgium; ^6^ National Plant Genetic Resources Laboratory Institute of Plant Breeding College of Agriculture and Food Science University of the Philippines Laguna Philippines; ^7^ International Institute of Tropical Agriculture c/o Nelson Mandela African Institution of Science and Technology Arusha Tanzania

**Keywords:** Botanic gardens, crop wild relatives, gap detection, seed germination

## Abstract

1. Ecologically meaningful seed germination experiments are constrained by access to seeds and relevant environments for testing at the same time. This is particularly the case when research is carried out far from the native area of the studied species.
2. Here, we demonstrate an alternative—the use of glasshouses in botanic gardens as simulated‐natural habitats to extend the ecological interpretation of germination studies. Our focal taxa were banana crop wild relatives (*Musa acuminata* subsp. *burmannica*, *Musa acuminata* subsp. *siamea*, and *Musa balbisiana*), native to tropical and subtropical South‐East Asia. Tests were carried out in Belgium, where we performed germination tests in relation to foliage‐shading/exposure to solar radiation and seed burial depth, as well as seed survival and dormancy release in the soil. We calibrated the interpretation of these studies by also conducting an experiment in a seminatural habitat in a species native range (*M. balbisiana—*Los Baños, the Philippines), where we tested germination responses to exposure to sun/shade. Using temperature data loggers, we determined temperature dynamics suitable for germination in both these settings.
3. In these seminatural and simulated‐natural habitats, seeds germinated in response to exposure to direct solar radiation. Seed burial depth had a significant but marginal effect by comparison, even when seeds were buried to 7 cm in the soil. Temperatures at sun‐exposed compared with shaded environments differed by only a few degrees Celsius. Maximum temperature of the period prior to germination was the most significant contributor to germination responses and germination increased linearly above a threshold of 23℃ to the maximum temperature in the soil (in simulated‐natural habitats) of 35℃.
4. Glasshouses can provide useful environments to aid interpretation of seed germination responses to environmental niches.

## Graphical abstract

Seeds of wild banana species germinate in response to subtle changes in temperature dynamics as found in direct exposure to the sun rather than in shade and are less tuned to temperature changes associated with burial depth. Results were obtained from experiments in environments used as proxies to natural habitats (glasshouses and a plant nursery). Methods are presented to aid interpretation of seed germination studies when access to natural environments is not possible.

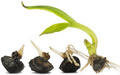

## 1 INTRODUCTION

Ideally, seed germination ecology studies are carried out in both natural habitats (NHs) and laboratory conditions (LCs) (Baskin & Baskin, 2014). This allows variables affecting germination to be clearly identified and ecologically interpreted. Interpretation is usually made in relation to spatial and temporal niches in NHs, or perhaps seminatural habitats (Semi‐NHs) (Table 1).

**TABLE 1:** Descriptions of environments for seed germination studies

Name	Definition	Germination example	Amount of control in experimental set‐up	Ability to ecologically interpret results to natural habitats
Natural habitat (NH)	Areas composed of viable assemblages of plant and/or animal species of largely native origin and/or where human activity had not essentially modified an area's primary ecological functions and species composition.^a^	Dinsdale et al. (2000)	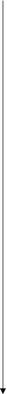	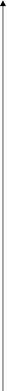
Semi‐natural habitat (Semi‐NH)	Ecological assemblages that have been substantially modified in their composition, balance or function by human activities.^a^	Stephens et al. (2012)		
Simulated natural habitat (Simulated‐NH)	A wholly constructed environment, made to resemble a NH			
Laboratory conditions (LC)	An un‐natural environment, variables are clearly defined and controlled	Mattana et al. (2016)		

^a^ European Investment Bank (2018).

In LC germination experiments, such as those using incubators, most variables are kept constant (e.g., light intensity, sowing medium, water availability, timing of diurnal temperature cycle), and one or two are manipulated with a few combinations (e.g., three or four temperatures of diurnal cycles). Researchers select the conditions of variables based on knowledge from NH microclimates, but these are notoriously difficult to exactly define (e.g., Dinsdale et al., 2000). Indeed, important variables may inadvertently be omitted from experimental designs. Interpretation of LC experiments in an ecologically meaningful way is difficult because it will be based on many assumptions, both in selecting variables to test and extrapolating interpretation to NHs—especially if NHs have not been adequately studied.

By contrast, in NH experiments many often unknown variables interplay, one or two of which may be controlled. If researchers were to control all combinations of NH variables in LCs, they would soon run out of seeds, time, and space. Interpretation of NH experiments depends on dynamically recorded variables that cannot be well controlled, some even being irrelevant to germination ecology. It goes without saying that NH experiments can only truly be performed in regions where the plant is native, whereas LC experiments can be carried out anywhere with suitable equipment.

Alternatives to NH and LC are seminatural habitats (Semi‐NHs) or simulated‐natural environments (Simulated‐NHs). Examples of Semi‐NHs include fields or farm edges, in or close to a species' native region. The term simulated‐natural environment (or habitat as used here) was used by Kaeberlein et al. (2002) to define the environment of natural seawater and sediment the authors placed in aquariums to culture previously “uncultivable” marine microorganisms. Such Simulated‐NHs are less controlled than LCs but allow better interpretation of findings; they may include important factors that are not well understood or known and so may inadvertently be omitted from LCs. Glasshouses, such as those in botanic gardens, are examples of Simulated‐NHs, as they mimic NHs and variables are not well under control.

In botanic gardens, living collections are often arranged according to geographic plant communities, each compartment or grouping representing a pseudo‐ or Simulated‐NH. These living collections are a valuable resource in studying plant ecology, particularly when NHs are challenging to access (Perez et al., 2019). Many botanic gardens also hold seed banks (469 gardens) or carry out seed or spore research (155 gardens) (BGCI, 2021). As botanic gardens are biased toward temperate regions in the Northern Hemisphere (Mounce et al., 2017), there is opportunity to enhance interpretation of seed germination studies using botanic gardens as Simulated‐NHs when it is not possible to do so in native regions (Faraji & Karimi, 2020).

**FIGURE 1:**
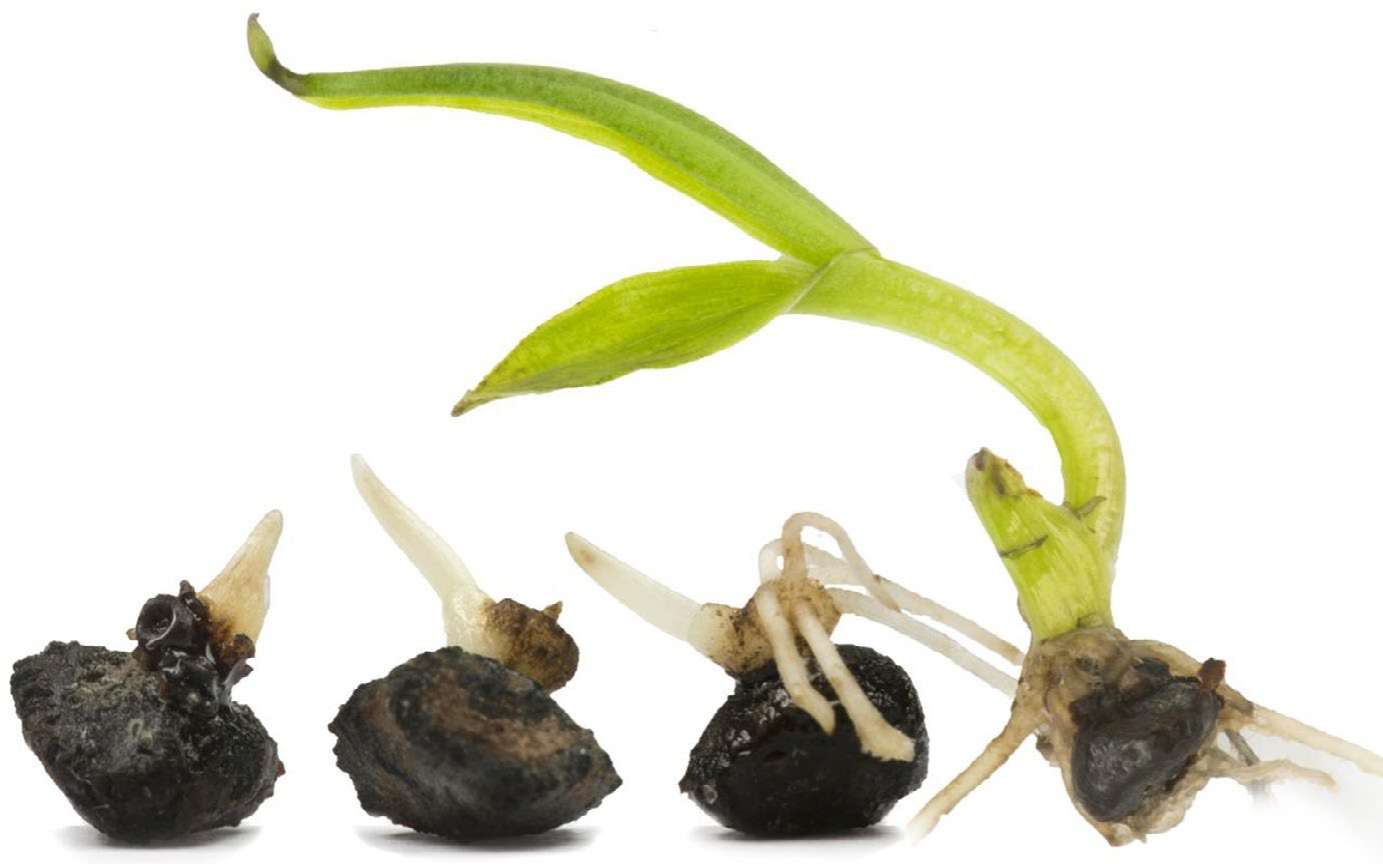
*Musa acuminata* seed during germination (image courtesy of Pablo Gomez Barreiro, Royal Botanic Gardens Kew)

Wild banana species (*Musa* L.) are native to tropical and subtropical Asia to the western Pacific (Govaerts & Häkkinen, 2006). Their fruit contains many hard dark seeds, 3–7 mm in diameter (Chin, 1996). The conditions for germination are not well understood, and germination is notoriously inconsistent and often very low (Figure 1) (Kallow et al., 2020; Panis et al., 2020; Singh et al., 2021). LC experiments show a requirement for alternating temperatures (Kallow et al., 2021; Stotzky & Cox, 1962), but no NH experiments have been executed to interpret this. For instance, is this requirement a gap or depth detection mechanism affected by microclimates? And do species respond differently?

Understanding seed germination ecology of wild bananas is not only of ecological interest, it is also important for global food security. Seed banking crop wild relatives efficiently protects genetic material and makes it available for phenotyping and breeding (Dempewolf et al., 2017). It is also included UN Sustainable Development Target 2.5 (UN General Assembly, 2015). Optimized germination is a vital component of seed bank management and breeding—without it, viability is difficult to monitor and access to plants for research and breeding is constrained (Amah et al., 2020; Batte et al., 2019; FAO, 2014).

In the present study, we examined germination responses in a Semi‐NH and Simulated‐NHs of the two primary crop wild relatives of banana: *Musa acuminata* (subsp. *siamea* N.W. Simmonds and subsp. *burmanicca* N.W. Simmonds) and *M. balbisiana* Colla (De Langhe et al., 2009). Specifically, we aimed to answer the following questions that cannot be answered in LCs: (a) What environments stimulate or inhibit *Musa* germination? (b) Are *Musa* seeds dormant, and if so, how is this broken in the environment? (c) Can *Musa* seeds remain viable in the soil?

## 2 MATERIALS AND METHODS

### 2.1 Seminatural habitat (nursery, the Philippines)

#### 2.1.1 Plant material

We collected a bunch (an infructescence) of *Musa balbisiana* (accession GB61996; Table 2) containing seeds from the field GenBank at the National Plant Genetic Resources Laboratory (NPGRL), Institute of Plant Breeding, University of the Philippines, Los Baños. Seeds were extracted by opening fruit and washing seeds in flowing water to remove all pulp. Seeds were then left on a tray in the laboratory to surface dry for 7 days prior to sowing.

#### 2.1.2 Solar radiation and substrate

We used the nursery of the NPGRL (latitude 14.153, longitude 121.262), as a Semi‐NH for germination testing. We used locations either exposed to solar radiation (“sun”)—only lightly shaded in the fine screen house or without direct exposure to solar radiation (“shade”)—in an open‐sided cabinet covered at the top also in the screen house. We sowed seeds in plastic trays (100 × 40 × 10 cm), using two types of substrate (clay loam soil and fine sand), and covered seeds with 5 mm of substrate. Two replicates of 200 seeds were used for each treatment combination. We recorded the temperature and relative humidity (RH), every 20 min for 55 days in sowing locations using data loggers (Tinytag View 2, Gemini Data Loggers). Trays were watered daily, and emergent seedlings were recorded and removed weekly. The test was concluded after 55 days.

### 2.2 Simulated‐natural environment (glasshouse compartments, Belgium)

#### 2.2.1 Plant material

We studied germination responses in Simulated‐NHs in relation to foliage‐shading and seed burial depth using two *Musa* species (total three taxa; Table 2). Seeds were selected from the collection of Bioversity International/KU Leuven (Leuven, Belgium) and were supplied for scientific use with a phytosanitary and origin certificate from China (Hainan), Guadeloupe, and Nigeria. Seeds were from open‐pollinated accessions in living collections and were air shipped from source to Leuven as complete bunches, where they were extracted as described above, apart from bal106 which were provided as extracted and cleaned seeds transported to Leuven, Belgium. After ambient drying, seeds were placed in the refrigerator (5°C) in paper bags, until 2019 when they were sealed in aluminum bags and stored in the refrigerator at approximately 6% moisture content, fresh weight basis. Viability was assessed prior to sowing (both in 2019 and 2020) by embryo rescue (ER)—germinating embryos extracted from the rest of the seed in vitro, to remove dormancy (method described by Kallow et al., 2020).

**TABLE 2:** Accessions used for germination experiments in simulated‐natural environments, V = viability percentage from embryo rescue tests in 2019 and 2020

Accession	Taxa	Source	Native distribution^a^	Year collected	V_2019_	V_2020_	Experiment
GB61996	*M. balbisiana*	the Philippines	Trop. & Subtrop. Asia	2019	NA	NA	Semi‐NH
bur60	*M. acuminata* subsp. *burmannica*	Guadeloupe	SW. India, China (S. Yunnan) to Indo‐China	2014	90	17	Simulated‐NH
sia61‐63	*M. acuminata* subsp. *siamea*	Guadeloupe	Indo‐China to N. Pen. Malaysia	2014	33	10	Simulated‐NH
bal106	*M. balbisiana*	China (Hainan)	Trop. & Subtrop. Asia	2017	87	58	Simulated‐NH
bal115	*M. balbisiana*	Nigeria	Trop. & Subtrop. Asia	2019	100	88	Simulated‐NH

^a^ Govaerts and Häkkinen (2006).

#### 2.2.2 Foliage‐shading and seed burial depth

We selected a total of six Simulated‐NHs for germination tests. Five were in three compartments of Meise Botanic Gardens glasshouse, Belgium (latitude 50.925, longitude 4.330), and one in the full‐ground glasshouse of KU Leuven, Belgium (latitude 50.860, longitude 4.680). Simulated‐NHs were selected to represent various heating regimes in different compartments and levels of foliage‐shading/exposure to solar radiation (Table 3). Compartments were selected because living banana specimens were already growing in them and heating regimes represented ranges found in species distributions (Kallow et al., 2021; Mertens et al., 2021), Semi‐NH microclimates (see Section 3), and previous LC studies (Kallow et al., 2021; Stotzky & Cox, 1962; Stotzky et al., 1961).

**TABLE 3:** Summary heating regimes of glasshouse compartments used, temperatures are thermostat temperature, below which compartments were heated

Compartment name	Institution	Exposure	Day (°C)	Night (°C)
Leuven	KU Leuven	Exposed	15	15
Mabundu	Meise Botanic Gardens	Exposed	25	25
Tropical	Meise Botanic Gardens	Shaded	20	18
Tropical	Meise Botanic Gardens	Exposed	20	18
Spring	Meise Botanic Gardens	Shaded	10	8
Spring	Meise Botanic Gardens	Exposed	10	8

Seeds were sown in square plastic pots (9 × 9 × 10 cm), at two burial depths (1 and 7 cm from the surface), selected to represent the expected depth of the majority of seeds in NHs (Dalling et al., 1998). Two replicates of 30 seeds of each accession were sown in separate pots in potting compost (Peltracom, composition: 70% white peat and 30% black peat; pH: 5.5– 6.5; particle size: 0–10 mm). Pots were then buried to be level with soil surface. Data loggers (Tinytag Transit 2 TG4080, Gemini Data Loggers) were buried at each location and burial depth. Loggers were set to record temperatures every 20 min. Additionally in 2020, loggers (HOBO Pendant MX2202, Onset) were placed at the soil surface to record light intensity and surface temperature. Germination was monitored weekly, and emergent seedlings were recorded and removed. Seeds were sown in early March 2019, and again in early March 2020, this is the end of the winter/start of the spring season in Belgium (mean outdoor temperature 6.4℃, climate‐data.org). The experiment was concluded in March 2021.

#### 2.2.3 Dormancy and stratification in the soil

To assess dormancy and dormancy loss in the soil (stratification), seeds from the two *M. balbisiana* accessions (bal106 and bal115) were incubated at alternating 35℃ in the light for 6 hr and 20℃ in the dark for 18 hr (based on Kallow et al., 2021; Stotzky et al., 1961). Additionally, seeds were buried at two of the cooler Simulated‐NHs (spring/exposed and Leuven/exposed) in March 2019 and exhumed each month for a total of 3 months and placed in the incubator conditions described. Seeds were enclosed in small nylon mesh bags and were buried at 7 cm depth in March 2019. Two replicates of 30 seeds were used for each treatment and accession. For incubation, seeds were sown on moist sand (50 g fine sand, 14 ml deionized water) in Petri dishes (9 cm diameter), sealed in plastic bags, at Meise Botanic Garden. Germinated seedlings of these were recorded and removed weekly for 2 months. Germination was counted as radicle emergence to 2 mm.

#### 2.2.4 Survival in the soil

**FIGURE 2:**
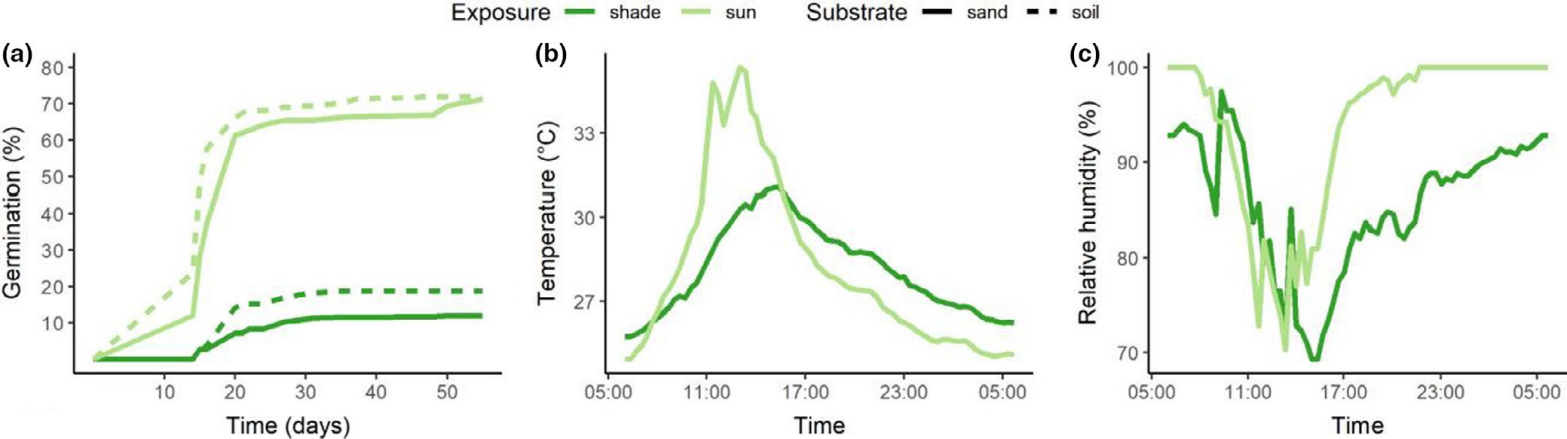
(a) Cumulative germination of *Musa balbisiana* seeds sown in the seminatural habitat (nursery, Los Baños, the Philippines), seeds were sown in the sun and shade, and in sand and soil (*n* = 200, two replicates, mean values shown); (b) typical diurnal temperature; and (c) relative humidity profiles logged at sowing site during germination test (Los Baños, the Philippines, 16 November 2019)

At the end of the experiment, seeds planted at shaded Simulated‐NHs (locations that showed no emergent seedlings in results) were extracted from pots and tested for viability using a tetrazolium chloride staining test or by incubation. Seeds sown in both 2019 and 2020 were used. Pots containing seeds were removed from compartments, seeds were separated from compost by sieving under running water. Extracted seeds from the first replicate of each treatment were tested for viability with tetrazolium chloride (TTC) with a maximum of 20 seeds. The TTC tests were carried out on embryos carefully extracted from seeds, using 0.5% TTC solution buffered to pH 7 (method described by Kallow et al., 2021), incubated at 24℃ for 24 hr in the dark. Extracted seeds from the second replicate were sowed in Petri dishes on top of moist sterilized potting compost (Peltracom, composition: 70% white peat and 30% black peat; pH: 5.5–6.5; particle size: 0–10 mm) and placed in an incubator at a 24‐hr cycled temperature pattern (based on temperature readings from Los Baños, the Philippines—sun‐exposed site; Figure 2a). Seeds in the incubator were monitored every 2 weeks, for a maximum of 5 weeks. If very few seeds were extracted from the soil for a replicate, TTC tests were prioritized above incubator germination tests.

### 2.3 Data analysis

We calculated summary indices for germination tests using the *GerminaR* package in R (Lozano‐Isla et al., 2019). These included final germination percentage (GRP, %) (Labouriau & Valadares, 1983), mean germination time (MGT, days) (Czabator, 1962), and synchronization index (SYN, simultaneous germination within a replicate = 1, no overlap between seeds of a replicate = 0) (Primack, 1980; Ranal & Santana, 2006).

We summarized data logger readings to extract the mean, maximum, minimum, and range (maximum–minimum) during experimental “cue periods.” In Semi‐NH, this was the whole experimental period (55 days). In Simulated‐NHs for each year, we summarized data filtered to include readings during “cue periods”—these were a period prior to the MGT of each replicate, set differently for shallow and deep sowing based on the mean MGT for shallow and deep sowing (shallow = 32 days, deep = 39 days). For locations with no germination, we used the first 39 days after sowing. We summarized logger readings for cue periods by calculating mean, maximum, minimum, range temperatures, and light intensity readings (in 2020). We removed light intensity readings in the dark (<40 lux), to account for changes in day/night length during seasons and did not calculate minimum light intensity (night time).

We then carried out redundancy analysis (RDA) of summarized logger data in cue periods, scaled to unit variance, against a corresponding matrix of factorial variables (species, compartment, exposure, and depth) using the *vegan* R package (Oksanen et al., 2019). The minimum adequate RDA was found by comparison of Akaike information criterion (AIC), by adding variables to the minimum model.

Final germination, for each experiment, was assessed using counts of germinated seeds at the end of experiments against seeds that did not germinate, thus accounting for sample size variance. Sample sizes were adjusted in the analysis to only include viable seeds, estimated from ER results of that year, using the formula:

adjusted sample=seeds sown×viability of year sown

These data were used in generalized linear modeling (GLMs) of binomial data with logit link function. If overdispersion was present in binomial GLMs, we used quasibinomial error structure. Minimum adequate models (MAM) were produced by removing variables from maximum models after comparisons with ANOVA and chi‐square test. Estimated marginal means were calculated from models and used for post hoc analysis using the *emmeans* R package (Lenth, 2020). Additionally, we used GLMs for factorial variables on MGT and SYN using gamma error structure. We assessed the effect of environmental variables in cue periods on binomial germination outcomes using GLMs as described. Following this, we tested variables for breakpoints to assess temperature thresholds or optimums. We did this on GLMs produced for each microclimate variable separately by using the algorithm with bootstrapping in the *segmented* R package (Muggeo, 2017). We used starting points estimated by plotting GRP against microclimate variables and trend lines using the nonlinear regression method Loess.

We compared viability of seed survival of seeds exhumed after 1–2 years in the soil from the incubation test, TTC test, and maximum achieved from Simulated‐NHs, against the original ER viability. We again used a binomial GLM for this, as described, and post hoc contrasts against original ER with Dunnett tests. All analyses were performed in R (R Core Team, 2020).

## 3 RESULTS

### 3.1 Effects of exposure to solar radiation and substrate on seed germination

In the Semi‐NH, exposure to direct solar radiation (sun) rather than shade significantly increased germination rates (*z* = 20.963, *p* < .001; Figure 2a). There was no significant effect of substrate on germination outcome according to the GLM. Final germination percentage in the sun was 72% (soil) and 71% (sand) whereas in the shade it was 19% (soil) and 12% (sand).

The MGT in the shade was 22 ± 2 days (mean, standard deviation, used hereafter), whereas in the sun it was slightly faster germinating in 18 ± 2 days, albeit only few seeds germinated in the shade. Synchronization index in the sun was 0.25 ± 0.10 and in the shade 0.27 ± 0.07.

Mean daily temperature in sun and shaded exposure was identical, but standard deviation was approximately 1℃ greater in the sun (27.3 ± 2.8℃ sun, 27.3 ± 1.9℃ shade; Figure 2b, Table S1). Most notably, this relates to warmer maximum temperatures in the day of around 4℃ in the sun. Mean diurnal range in the sun was therefore also greater (8.9℃ sun, 5.1℃ shade). Humidity in the sun and the shade was broadly similar, ranging diurnally between 75% and 100% RH (Figure 2c), a function of temperature.

### 3.2 Effects of foliage‐shading and seed burial depth on seed germination

#### 3.2.1 Microclimate in simulated‐natural habitats

**FIGURE 3:**
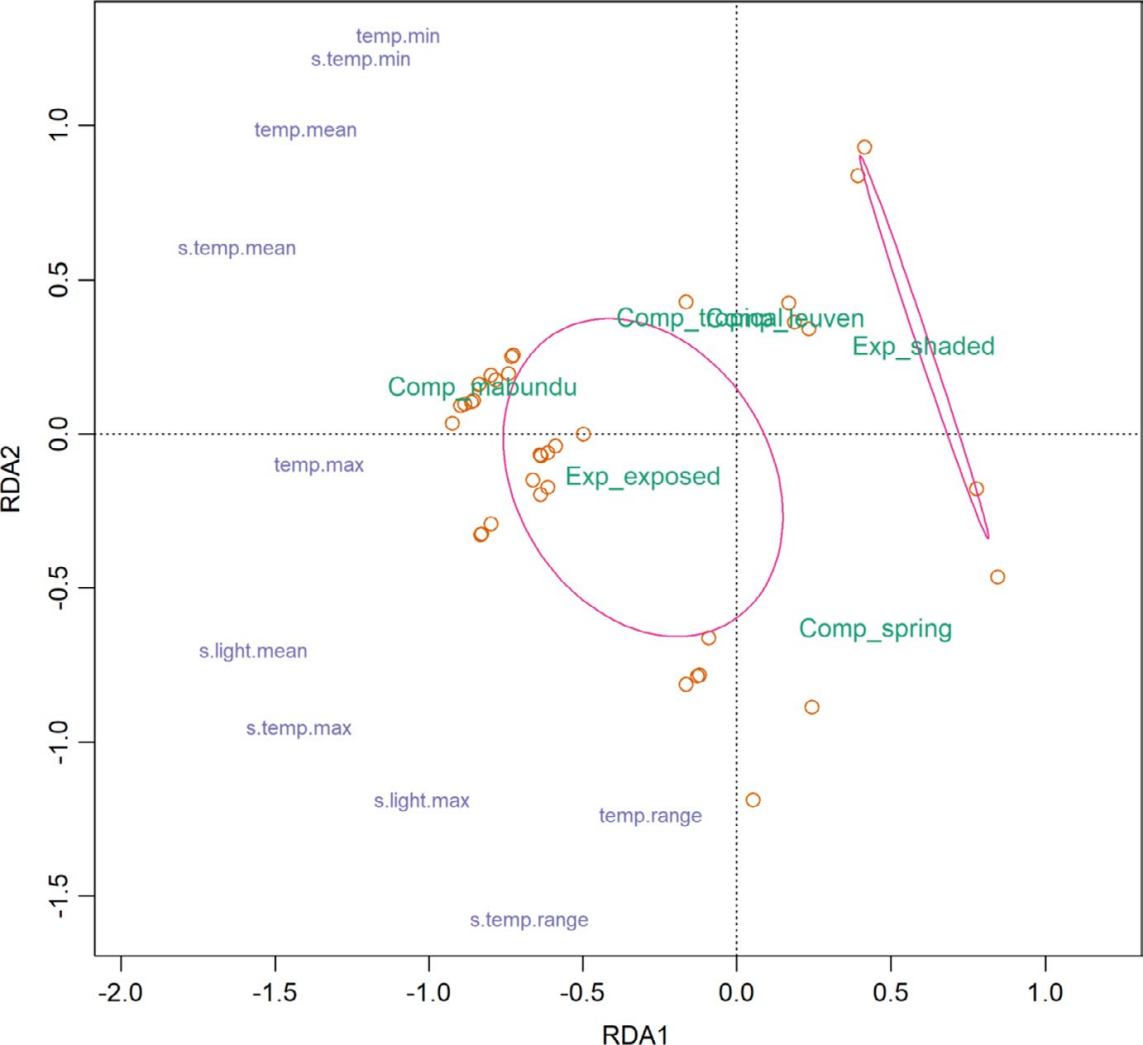
Redundancy analysis ordination of microclimates used as simulated‐natural habitats in glasshouses for seed germination experiments (*r* ^2^ = 0.88), ellipses are exposed and shaded sites (confidence limit = 0.95), green text is explanatory factors (Comp = compartment, Exp = level of exposure to sun), blue text are microclimate variables achieved from data loggers at site (s = from data logger at soil surface, temp = temperature)

Cue period microclimate variables were constrained by the factors glasshouse compartment and exposure in the minimum adequate RDA, and notably not by seed burial depth or species (*r* ^2^ = 0.88; Figure 3). Exposure had the greatest influence on the microclimate RDA (*df* = 1, *F* = 262.15, *p* < .001), and glasshouse compartment had greater variance (*df* = 3, *F* = 144. *p* < .001). Unsurprisingly, compartments with higher thermostat temperature thresholds (Table 3) were clustered with higher mean and minimum temperatures. These clusters did not include variables relating to extremes, that is, maximum temperatures, temperature ranges, and soil surface values (for temperature and light). These were clustered separately and correspond to exposed microclimates. Shaded microclimates varied less than exposed (hence the different shapes of ellipses in Figure 3).

Shaded microclimates obviously received less light intensity (731 ± 240 lux shaded, 4,491 ± 1,621 lux exposed) (Figure S1) and had a lower maximum temperature (25.8 ± 1.0℃ shaded, 40.7 ± 7.1℃ exposed). In the soil, the maximum temperature, again, was less at shaded sites compared with exposed sites (19.5 ± 2.2℃ shaded, 27.1 ± 4.6℃ exposed). The relationship between light intensity and temperature at the soil surface was asymptotic, above 2,000 lux there was very little increase in temperature (Figure S2).

#### 3.2.2 Effects of microclimate factors on seed germination

**FIGURE 4:**
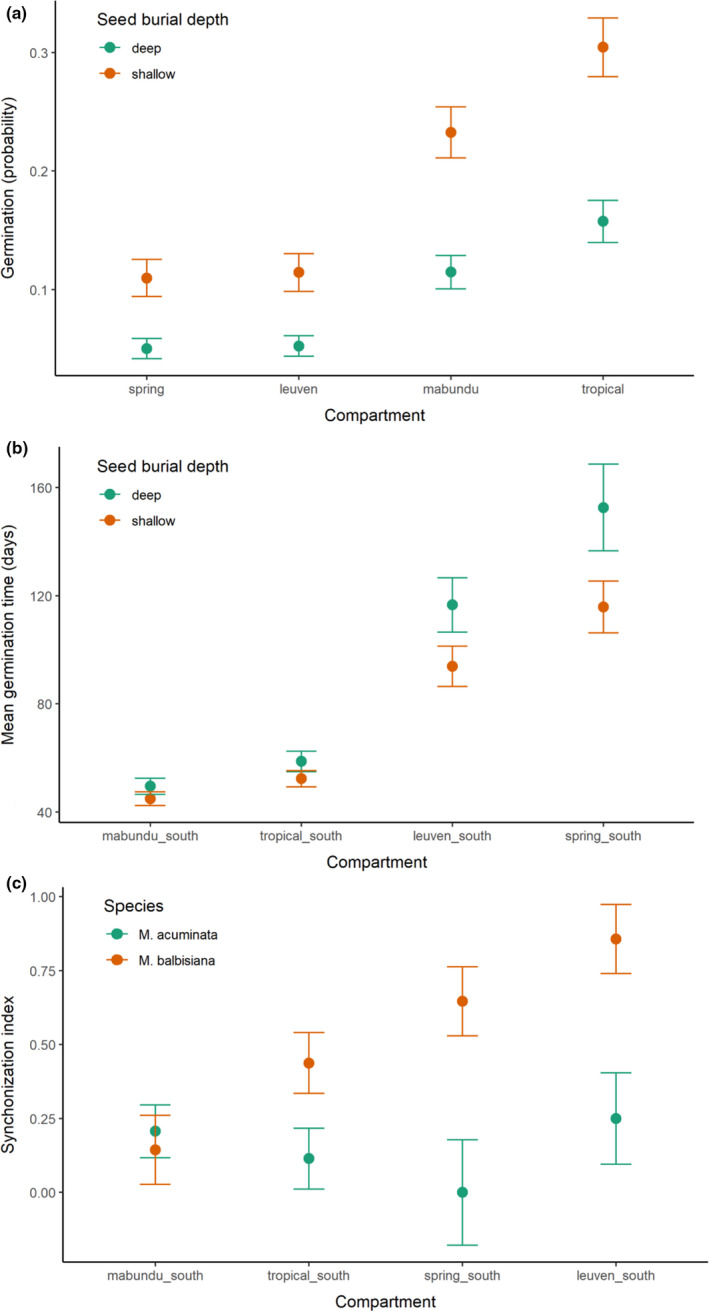
Estimated marginal means of minimal adequate GLMs on germination of *Musa* seeds buried in Simulated‐NHs in Belgium (a) binomial germination outcome; (b) mean germination time; (c) synchronization index

In Simulated‐NHs, no seeds germinated in any shaded microclimates. These were then excluded from statistical analysis. Compartment and depth remained in the MAM, species and year were excluded after testing (Figure 4a). Seeds had significantly higher probability of germinating in the two compartments that have a higher heating regime (compartments Mabundu, *z* = 4.531, *p* < .001, and Tropical *z* = 6.586, *p* < .001). Across all glasshouse compartments, shallower buried seeds were more likely to germinate (*z* = 6.491, *p* < .001).

Seedling emergence happened sooner after sowing in the compartments with warmer heating regimes (Mabundu, *t* = 8.557, *p* < .001 and Tropical 6.805, *p* = <.001; Figure 4b). Predictably, shallow buried seeds at 1 cm emerged quicker than those buried at 7 cm (*t* = 2.657, *p* = .010). There was no effect of species on time to germination (seedling emergence), so this was excluded from the model, leaving compartment and burial depth.

Synchronization was significantly greater in the cooler compartments (spring and Leuven) for *M. balbisiana* (Figure 4c), particularly in the pairwise contrast between Leuven and Mabundu (*z* = 3.110, *p* = .010).

#### 3.2.3 Effects of microclimate variables on seed germination

**FIGURE 5:**
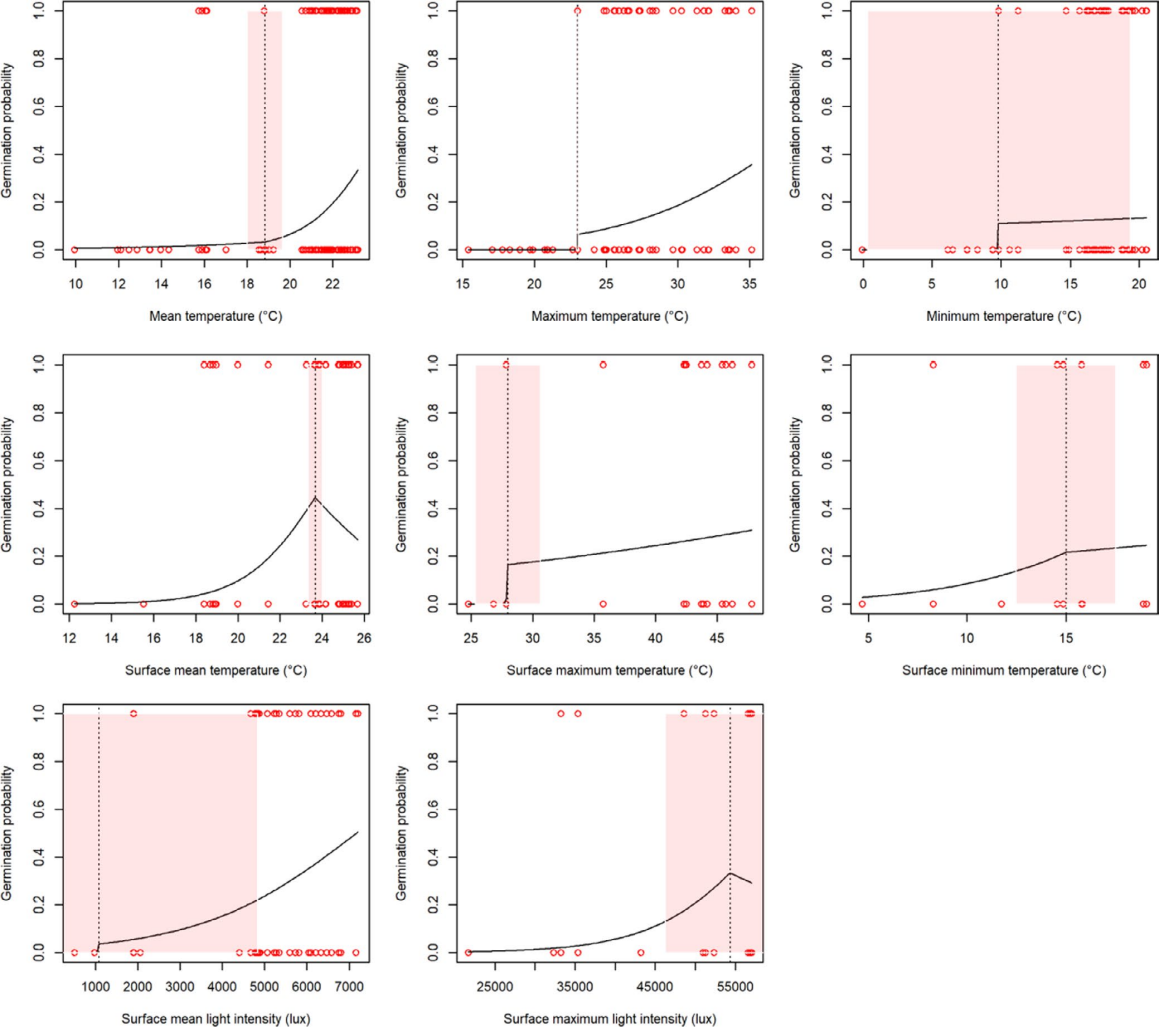
Segmented GLMs of binomial germination outcomes back‐transformed to the response probability scale as response to microclimate variables in Simulated‐NHs, dashed lines are breakpoints, pink shading is standard error (0.95), and residuals are red circles

The effect of each microclimate variable on final germination percentage was visualized (Figure S3). These were then used to estimate breakpoints on GLMs produced from binomial germination outcomes (Figure 5). Results of this show temperatures (at seed burial level) operate a threshold mechanism, above which germination increases linearly (on the logit scale), within the limits of temperatures achieved in Simulated‐NHs. Therefore, for germination to occur mean soil temperature needs to be above 19℃, maximum temperature must be above 23℃, but minimum temperature has much less of an effect. At the soil surface, the effect of mean temperature is positively linear to an optimal at 24℃, after which germination is reduced. The effect of maximum temperature is positive above a threshold of 28℃. Germination increases with soil surface minimum temperature to 15℃, then the positive slope is less steep. Soil warming relates to light intensity asymptotically (as described above), germination increased above 1,076 lux (mean). In relation to maximum light intensity, germination increased to a breakpoint at 54,000 lux, after which the effect on germination is negative. Comparisons of AICs of these models (Table S2) show at seed burial level the maximum temperature is the best fit, at soil surface level mean temperature is the best fit, and mean light intensity is a better fit than the maximum value during cue periods.

### 3.3 Survival and dormancy loss in the soil

**FIGURE 6:**
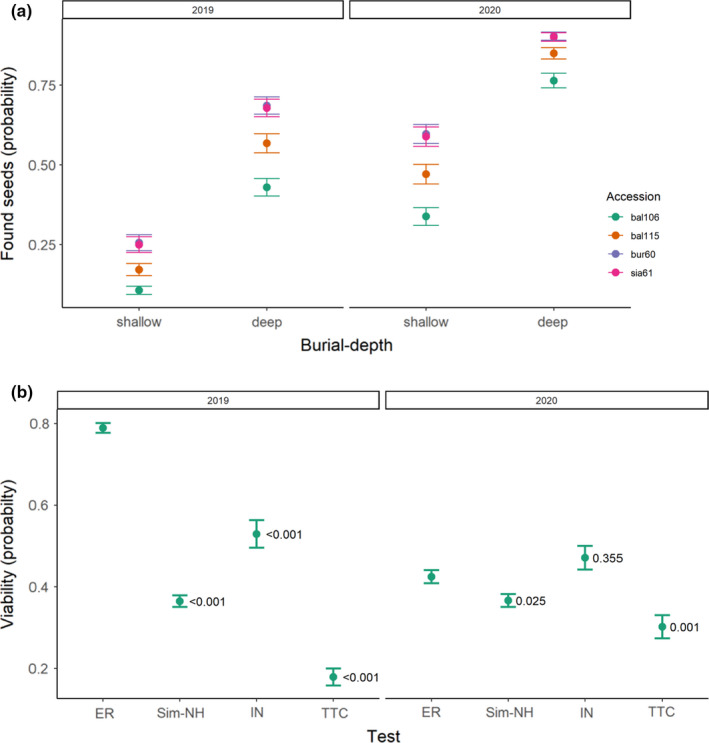
Seed survival in the soil; (a) probability of finding seeds in 2021 that were sown in 2019 and 2020 according to burial depth and accession; (b) viability of seeds found in 2021 using a germination test in an incubator (IN) and the tetrazolium chloride test (TTC), with reference to viability at the start of the experiment using embryo rescue techniques (ER), and maximum germination achieved in a Simulated‐NH; *p* values show contrasts (Dunnett test) against original viability (ER) for each year

Overall, 40% of seeds that were buried in the soil for 2 years and 68% for 1 year were successfully exhumed from the soil. The probability of finding seeds was modeled against year, burial depth, and accession (Figure 6a). Seed burial depth was the most important factor, and deep‐buried seeds were more likely to be successfully exhumed (*z* = 16.722, *p* < .001), followed by year (*z* = 13.379, <0.001). Surprisingly, seeds with low viability when sown had high probability of being exhumed (bur60 *z* = 7.242, *p* < .001, sia61 *z* = 7.025, *p* < .001).

Viability of exhumed seeds was tested by TTC and incubation. Viability tests from these, original ER and maximum in Simulated‐NHs, were modeled in a GLM (Figure 6b). In contrast to the results of the above, accession was excluded from the MAM, year remained. Contrasts were made per year against original ER. Viability (according to the incubator test) was not lost during 1 year in the soil. After 2 years in the soil, viability was reduced in both incubator and TTC tests. The TTC test consistently underestimated viability.

**FIGURE 7:**
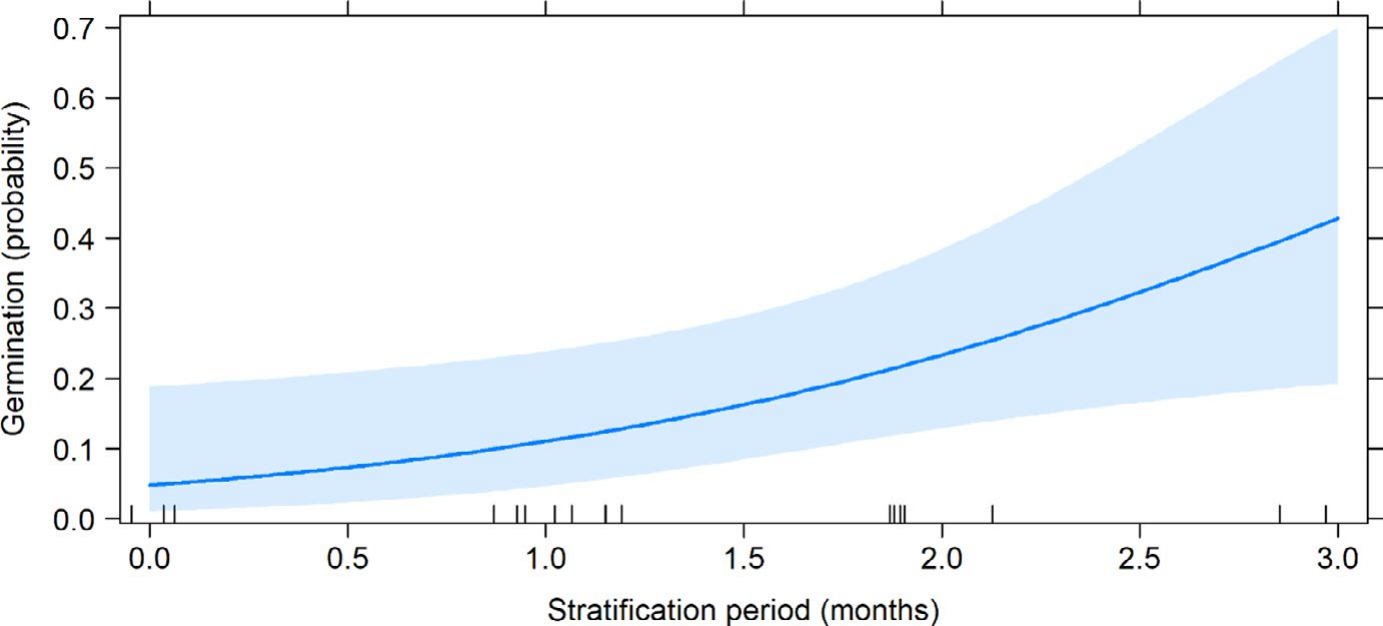
Germination probability in an incubator (at 35/20℃, light/dark, 18/6 hr cycled) of seeds buried and then exhumed from the soil for up to 3 months. Seeds were buried in the exposed Spring compartment

Seeds that were removed from storage and incubated under suitable conditions (20/35℃) displayed dormancy; that is, they did not germinate at all. However, if they were buried in the soil at 7 cm for up to 3 months, germination significantly increased to probability 0.4 (*t* = 2.518, *p* = .021; Figure 7).

## 4 DISCUSSION

### 4.1 Use of seminatural and simulated habitats for germination ecology experiments

In the present study, we used a Semi‐NH from the native range of *M. balbisiana*, and several Simulated‐NHs in glasshouse compartments located in a temperate region to examine wild banana seed germination ecology. We established that with such approximations of NHs, it is possible to link germination responses to ecological factors such as foliage‐shading and burial depth. Hence with this approach, we overcame limitations when access to experimental NHs was not possible, this allowed for greater ecological interpretation than with LCs alone.

#### 4.1.1 Temperature

We found that *Musa* seed germination is stimulated by exposure to the sun. It was the maximum part of the temperature fluctuation, in our results, that is most closely associated with germination. Above the threshold of 23℃, germination increased to a maximum at 35℃ (in Simulated‐NHs and 42℃ in Semi‐NHs) in exposed conditions. Our findings are broadly consistent with previous LC results, where optimal maximal and minimal temperature for germination were diurnal cycles of 35/18–20℃ respectively, for both *M*. *acuminata* and *M. balbisiana* (Kallow et al., 2021; Stotzky & Cox, 1962). Interactions between the elements of warming and cooling cycles play an important role in simulating germination.

#### 4.1.2 Light

One might think that as germination responses were directly associated with soil exposure to sun and, in Simulated‐NHs, light intensity, germination is stimulated by light. Additionally, we also found that seeds germinated to a greater extent from 1 cm compared with 7 cm. However, light waves cannot usually penetrate the soil to greater than 4–5 mm depth (depending on soil moisture and particle size), and not to any amount that can illicit germination responses to light‐sensitive seeds (Tester & Morris, 1987; Woolley & Stoller, 1978), but in our experiment, seeds germinated from a depth of 7 cm. We therefore infer that while *Musa* seed germination may be correlated with factors associated with light (light intensity, shallow burial), theses are correlations rather than causal, and it is temperature that regulates germination.

#### 4.1.3 Gap detection

*Musa* seed germination in response to sun exposure demonstrates adaptation to detect suitable niches for seedling establishment following disturbance in forest NHs. Conversely, inhibition of germination in shade is also an adaptation for seedling survival (Kos & Poschlod, 2007; Poschlod et al., 2013). *Musa* germination responses were sensitive to sun/shade even when microclimates were very similar. For instance, mean temperatures were the same and variance differed by only a few degrees in sun and shaded Semi‐NHs. Germination is therefore finely tuned to respond to microclimate such as that which would occur when a forest gap is formed (Pearson et al., 2002, 2003).

The effect of forest disturbance on temperature dynamics was studied by Hardwick et al. (2015). The authors measured soil (10 cm depth) and air temperature (1.5 m height) at three levels of forest disturbance in Borneo. Diurnal air temperatures in oil palm plantations (formerly forested) were around 7℃ higher at the hottest part of the cycle compared with old‐growth forests, and soil temperatures were around 3℃ warmer at this point and around 1℃ cooler in the night—these were similar to our results in Semi‐NHs. Temperatures in logged forests were somewhat in between these two, but more like old‐growth forest. One could imagine that even in small forest gaps, microclimates could therefore also vary considerably (Pearson et al., 2002). These responses are in line with adaptations of other disturbance‐adapted species that also require alternating temperature cycles rather than constant temperatures to germinate (Pearson et al., 2002; Seiwa et al., 2009; Vázquez‐Yanes & Orozco‐Segovia, 1982, 1993).

#### 4.1.4 Seed burial depth

When seeds were in the shade, burial depth made no difference to germination: they did not germinate irrespective of burial depth. In exposed sites, shallow buried seeds were more likely to germinate than deeply buried seeds. This was because air temperature dynamics are buffered by burial depth. For some species, sensitivity to alternating temperatures is an adaptation to detect burial depth (Thompson & Grime, 1983; Thompson et al., 1977). Further, Pearson et al. (2002) found, for large‐seeded species, diurnal temperature sensitivity was more likely related to forest gap size than seed burial depth. This was also the case for *Musa* (comparable to Pearson et al.'s large‐seed classification) in that exposure was by far the most significant factor in Simulated‐NHs, and burial depth was only secondary. For small seeds, it is important to detect burial depth as seedlings must reach the surface with small endosperm reserves; for larger seeds with greater nutrient reserves, this is less of a limiting factor for survival.

### 4.2 Survival and dormancy loss in the soil

We found seeds can persist and remain viable buried in the soil for at least 2 years. In fact, there was no loss of viability after 1 year, there was, however, loss of actual seeds. Seed loss was more pronounced with shallow burial, suggesting it is the result of predation or perhaps splashing causing seeds to be washed out of pots during watering, rather than decomposition. This is also supported by the fact that accessions with less viable seeds were more likely to be found‐ that is, seeds were not lost by decomposition of dead seeds.

For seeds adapted to disturbance, seed persistence in the soil seed bank is important. *Musa* clearly invest considerably in seed coat defenses (Graven et al., 1996), to survive the intense pressure present in the soil community (Dalling et al., 2011).

Not only do seeds persist in the soil, but also dormancy is reduced during this process. In our results, when seeds were warm stratified for 3 months, or when they were in the soil for a year, germination increased. A warm stratification requirement is in keeping with results from our previous study (Kallow et al., 2021). Although stratification was not required for freshly extracted *M. balbisiana* seeds in Semi‐NHs, implying drying induces secondary dormancy in *Musa* seeds, as proposed by Chin (1996).

There was greater germination synchronization in cooler Simulated‐NHs as seeds responded to threshold temperatures when sun was stronger during the summer. When temperatures were consistently warmer, synchronicity was reduced—this may again be a disturbance adaptation. We found this response more evident in *M. balbisiana* than in *M*. *acuminata*, suggesting it may also relate to seasonality as *M. balbisiana* has a large distribution that includes subtropical seasonal climates (Mertens et al., 2021).

### 4.3 Limitations

While in the present study we extended the ecological interpretation of seed germination experiments using botanical glasshouse environments as proxies for NHs, manipulation of glasshouse environments is constrained by the infrastructure available. Some environments are more challenging to simulate than others. For example, it is more difficult to simulate cool seasonal environments in a hot tropical climate than in the present study where tropical and subtropical environments were simulated in a temperate country adding heating and humidity. Infrastructure of this kind may not be available for many institutions, and on a large scale, it is expensive. Simple manipulations are of course possible, such as shade cloths, but this is not enough to create Simulated‐NHs in environments for focal species considerably removed from their NHs.

Additionally, with the approach presented here we are seeking to extend the ecological interpretation of seed germination experiments by performing then in environments closer to species NHs, but it is important to note that these are still removed from actual NHs and caution should be made when interpreting results. In addition to using Simulated‐NHs, we grounded our experiments using Semi‐NH, as well as previous experiments based on climate data (Kallow et al., 2021). However, additional factors may also play a role in germination that are not controlled for. These include artificial harvest, cleaning, shipping, and storage, as well as using seeds collected from non‐natural populations and environments in field collections in non‐native countries.

## 5 CONCLUSIONS

Studying germination ecology has intrinsic challenges, possibly the biggest being access to seeds and experimental set‐ups in suitable conditions and timeframes. In the present study, we demonstrate an approach for dealing with such difficulties in studying tropical seed germination ecology, which is a challenge when researchers are outside of a plant's native region. Using Semi‐NHs and Simulated‐NHs, we found the following: (a) Foliage‐shading inhibits germination of nondormant seeds, and exposure to sun stimulates germination, this response is most closely associated with maximum temperature variation found under direct sunlight, this effect is marginally buffered by deep burial in the soil; (b) freshly extracted seeds are nondormant, but stored seeds lose their dormancy during burial in the soil; (c) *Musa* seeds remain viable in the soil for at least a year without any loss in viability. Thus, wild banana species are well adapted to exploit canopy gaps following disturbance.

## CONFLICT OF INTEREST

The authors declare that there is no conflict of interest associated with this article and research.

## AUTHOR CONTRIBUTIONS

**Simon Kallow:** Conceptualization (equal); Data curation (lead); Formal analysis (lead); Investigation (lead); Methodology (equal); Software (lead); Validation (lead); Visualization (lead); Writing‐original draft (lead); Writing‐review & editing (lead). **Katrijn Quaghebeur:** Investigation (supporting). **Bart Panis:** Conceptualization (equal); Funding acquisition (equal); Methodology (supporting); Resources (equal); Supervision (equal); Writing‐review & editing (equal). **Steven B. Janssens:** Conceptualization (equal); Funding acquisition (equal); Methodology (supporting); Resources (equal); Supervision (equal); Writing‐review & editing (equal). **John Dickie:** Funding acquisition (equal); Supervision (equal); Writing‐review & editing (equal). **Lavernee Gueco:** Resources (equal); Writing‐review & editing (equal). **Rony Swennen:** Supervision (equal); Writing‐review & editing (equal). **Filip Vandelook:** Conceptualization (equal); Methodology (equal); Resources (equal); Writing‐review & editing (equal).

### OPEN RESEARCH BADGES

This article has earned an Open Data Badge for making publicly available the digitally‐shareable data necessary to reproduce the reported results. The data is available at https://doi.org/10.6084/m9.figshare.14884470.v1.

## Supporting information

Supplementary Material

## Data Availability

All data available at Kallow, Simon (2021): Dataset: Using seminatural and simulated habitats for seed germination ecology of banana wild relatives. https://doi.org/10.6084/m9.figshare.14884470.v1.
