# Supplementary material for: Using seminatural and simulated habitats for seed germination ecology of banana wild relatives
Source: Ecol Evol. 2021 Oct 11;11(21):14644–57. doi: 10.1002/ece3.8152 (PMC8571623; doi:10.1002/ece3.8152)
Supplement: Supplementary file 1 — Supplementary Material [file ECE3-11-14644-s001.docx]

## Supplementary figures and tables

##
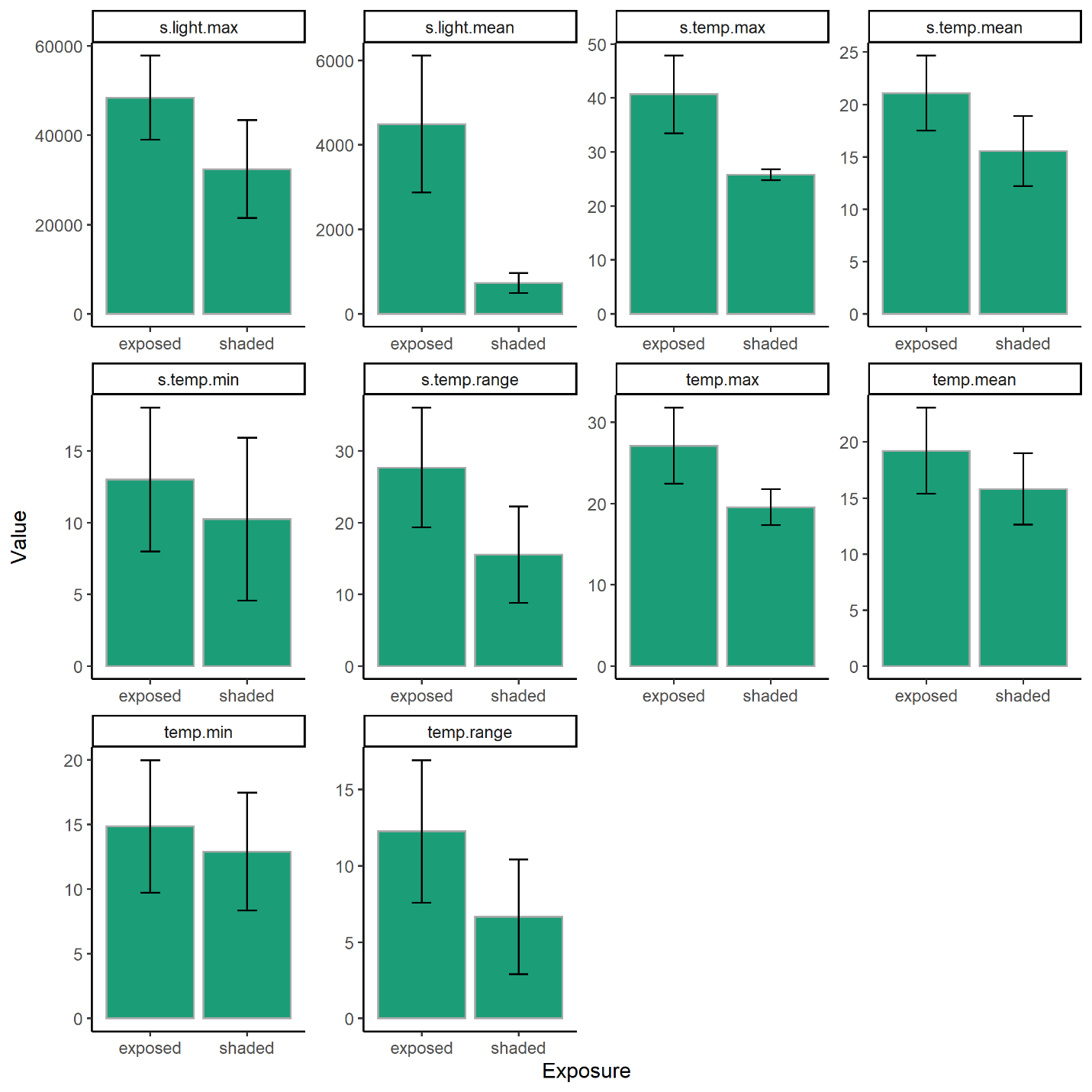


**Fig. S1.** Average and standard deviations of temperature and light intensity during cue periods of exposed and shaded environments, temperatures are °C, light intensity is lux.

##
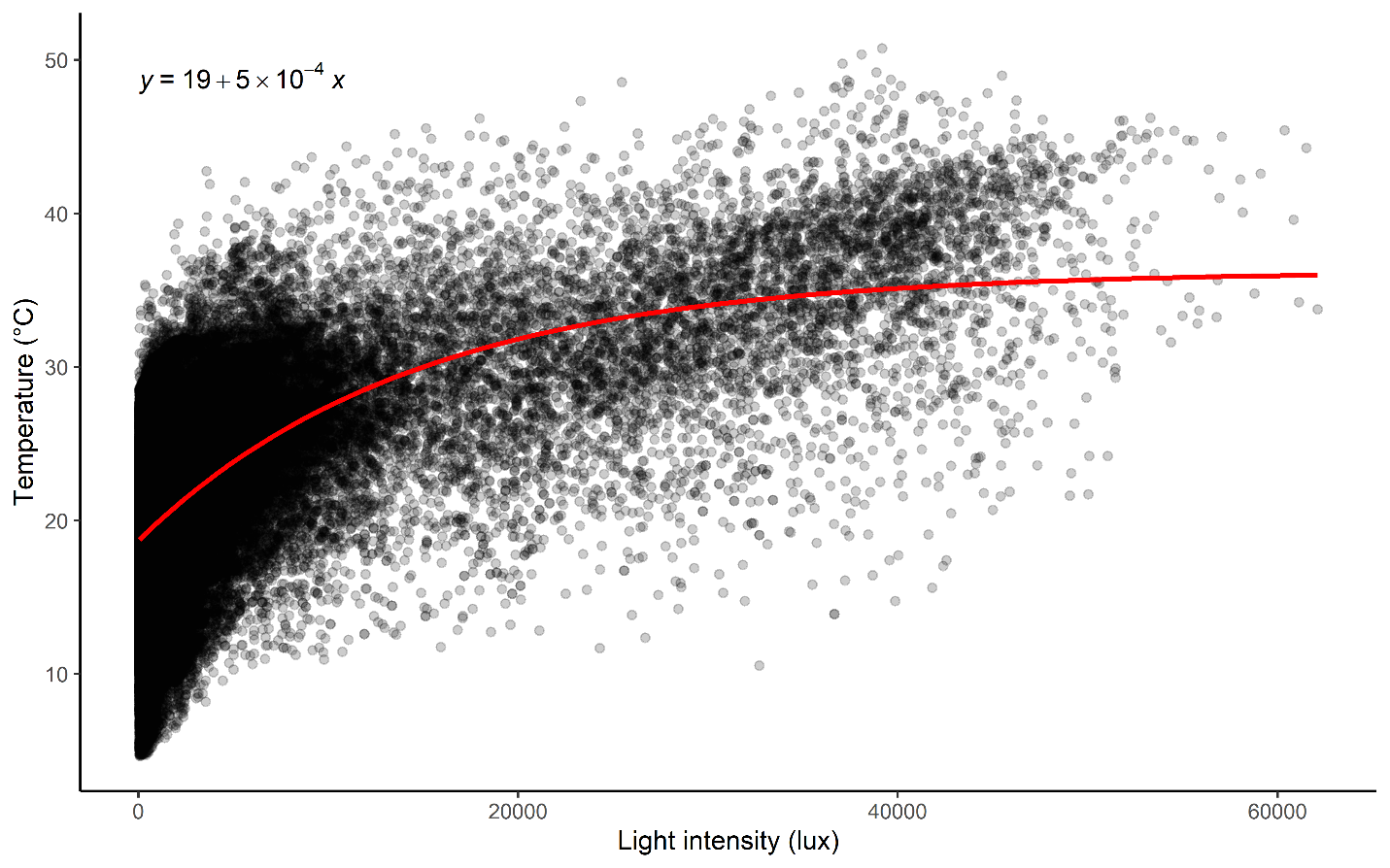


**Fig. S2.** Light intensity and temperature measured at simulated natural environments in glasshouses during germination tests; asymptotic non-linear regression shown (residual standard error 4.119 on 417,661 degrees of freedom).


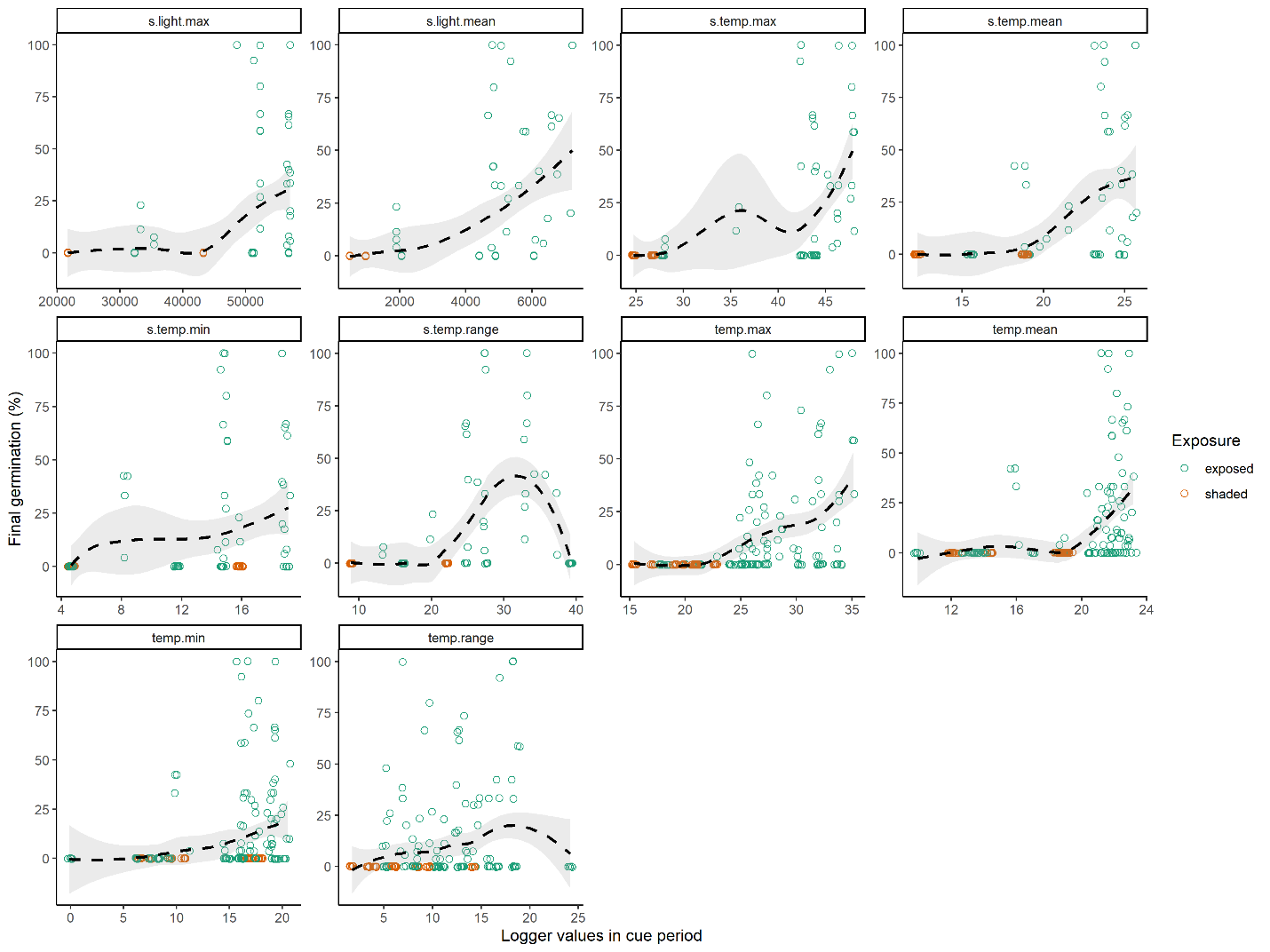


**Fig. S3.** Final germination percentages and microclimate variables from simulated-NHs, dashed trend lines plotted with Loess non-parameter regression, shaded area is 95% standard error, light variables are in lux, temperature are °C, s=value from logger at the soil surface, otherwise they are at seed burial depth.

**Table S1.** Summaries of the daily temperature and humidity recordings in the sun and shade at the location of the seed germination experiments. Values in brackets are standard deviations of means.

|  | Sun | | | | Shade | | | |
| --- | --- | --- | --- | --- | --- | --- | --- | --- |
|  | Temperature (°C) | | Relative humidity (%) | | Temperature (°C) | | Relative humidity (%) | |
| Daily | Night | Day | Night | Day | Night | Day | Night | Day |
| Mean | 24.5 (±0.8) | 33.4 (±3.6) | 81.3(±16.6) | 100 (±0.1) | 25.2 (±0.7) | 30.3 (±2.0) | 75.0 (±9.8) | 97.5 (±2.3) |
| Max | 26.2 | 42.2 | 100 | 100 | 26.7 | 38.5 | 94.3 | 100 |
| Min | 23.0 | 24.7 | 10.1 | 99.6 | 23.6 | 25.8 | 56.9 | 90.9 |

**Table S2.** Model fits of germination outcome in response to microclimate variables in simulated-NHs in segmented GLMs.

| Variable | AIC | Residual deviance | df |
| --- | --- | --- | --- |
| temp.mean | 1749 | 1741 | 3388 |
| temp.max | 1612 | 1604 | 3388 |
| temp.min | 1890 | 1882 | 3388 |
| s.temp.mean | 799 | 791 | 1220 |
| s.temp.max | 817 | 810 | 1220 |
| s.temp.min | 961 | 953 | 1220 |
| s.lightemp.mean | 803 | 796 | 1220 |
| s.lightemp.max | 887 | 880 | 1220 |
